# Associations of body composition and physical fitness with gestational diabetes and cardiovascular health in pregnancy: Results from the HealthyMoms trial

**DOI:** 10.1038/s41387-021-00158-z

**Published:** 2021-06-07

**Authors:** Pontus Henriksson, Johanna Sandborg, Emmie Söderström, Marja H. Leppänen, Victoria Snekkenes, Marie Blomberg, Francisco B. Ortega, Marie Löf

**Affiliations:** 1 Department of Health, Medicine and Caring Sciences, Linköping University, Linköping, Sweden; 2 Department of Biosciences and Nutrition, Karolinska Institutet, Huddinge, Stockholm Sweden; 3 Folkhälsan Research Center, Helsinki, Finland; 4 Faculty of Medicine, University of Helsinki, Helsinki, Finland; 5 Department of Obstetrics and Gynecology and Department of Biomedical and Clinical Sciences, Linköping University, Linköping, Sweden; 6 PROFITH (PROmoting FITness and Health through physical activity) Research Group, Department of Physical Education and Sports, Faculty of Sport Sciences, Research Institute of Sport and Health, University of Granada, Granada, Spain

**Keywords:** Cardiovascular diseases, Disease prevention, Public health, Risk factors

## Abstract

The aim of this study was to examine associations of body composition (fat mass index, % fat mass, fat-free mass index, body mass index) and physical fitness (cardiorespiratory fitness and handgrip strength) with gestational diabetes and cardiovascular health in early pregnancy. This cross-sectional study utilized baseline data (*n* = 303) collected in early pregnancy from the HealthyMoms trial. Body composition was measured using air-displacement plethysmography, cardiorespiratory fitness was assessed by means of the 6-min walk test and handgrip strength using a dynamometer. Logistic regression was used to estimate odds ratios (ORs) for gestational diabetes as well as high (defined as 1 SD above the mean) blood pressure, homeostatic model assessment for insulin resistance (HOMA-IR), and metabolic syndrome score (MetS score) per 1 SD increase in body composition and fitness variables. Fat mass index, % fat mass and body mass index were all strongly associated with gestational diabetes (ORs: 1.72–2.14, *P* ≤ 0.003), HOMA-IR (ORs: 3.01–3.80, *P* < 0.001), blood pressure (ORs: 1.81–2.05, *P* < 0.001) and MetS score (ORs: 3.29–3.71, *P* *<* 0.001). Associations with fat-free mass index were considerably weaker (ORs: 1.26–1.82, *P* = 0.001–0.15) and were strongly attenuated after adjustments for fat mass index (ORs: 0.88–1.54, *P* = 0.039–0.68). Finally, greater cardiorespiratory fitness was associated with lower risk of high HOMA-IR and MetS score (ORs: 0.57–0.63, *P*  ≤ 0.004) although these associations were attenuated when accounting for fat mass index (ORs: 1.08-1.11, *P* ≥ 0.61). In conclusion, accurately measured fat mass index or % fat mass were strongly associated with gestational diabetes risk and markers of cardiovascular health although associations were not stronger than the corresponding ones for body mass index. Fat-free mass index had only weak associations with gestational diabetes and cardiovascular health which support that the focus during clinical care would be on excess fat mass and not fat-free mass.

## Background

Pregnancy induces significant changes to the cardiovascular system^1^ and evidence demonstrates that the cardiovascular health during pregnancy is predictive of pregnancy outcomes as well as for long-term health outcomes postpartum^2^. For instance, gestational diabetes or hyperglycemia during pregnancy is associated with greater risk of adverse pregnancy outcomes such as preeclampsia, cesarean section and perinatal mortality^3,4^ as well as the long-term risk of type-2 diabetes^5^. Similarly, hypertensive disorders during pregnancy have been linked to maternal and infant mortality and morbidity^6^ and hypertension later in life^7^. Studies also suggest that clustering of individual cardiovascular risk factors such as glycemia, high blood pressure, high triglycerides and low high-density lipoprotein (HDL) cholesterol, all components in the metabolic syndrome (MetS), are more strongly associated with adverse pregnancy outcomes than single risk factors^8,9^. For instance, a recent study used the health-oriented ideal cardiovascular health framework by the American Heart Association^10^ to describe cardiovascular health and found that a composite score of five cardiovascular health metrics (glycemia, blood pressure, triglycerides, body mass index [BMI] and smoking) was associated with wider range of pregnancy outcomes than the individual metrics^9^. Altogether, the literature, e.g.^2–5,7^, support pregnancy as a unique opportunity to identify women at risk for future cardiovascular events and to provide prevention and treatment strategies to support long-term cardiovascular health^2^. However, despite the importance of favorable cardiovascular health during pregnancy, there are still gaps of knowledge regarding modifiable risk factors of poor cardiovascular health during pregnancy which may be of importance for effective preventive and treatment strategies.

Firstly, although obesity is a strong risk factor for gestational diabetes and other cardiovascular risk factors such as gestational hypertension and preeclampsia^11^, categorization of obesity is based on BMI which is a relatively poor marker of body fatness also in pregnancy^12^. Furthermore, BMI cannot differentiate between fat mass (FM) and fat-free mass (FFM), which may have different health effects^13^. The few studies that have examined body composition, using accurate methodology, in relation to cardiovascular health have generally reported positive associations of FM with glycemia and insulin resistance^14–16^. However, little is known whether the FFM of the body weight is related to cardiovascular health and the few previous studies have shown conflicting results^15,17^. Furthermore, it is not fully examined whether a state-of-the art body composition measure would convey cardiovascular health better than BMI which is widely used but also widely criticized^18^.

Secondly, cardiorespiratory fitness is considered a strong marker of health and has been linked to more favorable cardiovascular health in numerous studies of non-pregnant populations^19,20^. Furthermore, adequate cardiorespiratory fitness has also been found to attenuate the negative health effects of obesity^18,21^. However, to the best of our knowledge, no previous study has examined associations of physical fitness with cardiovascular health in pregnant women.

The aim of this study was therefore to examine associations of body composition and physical fitness with cardiovascular health (i.e. glycemia and gestational diabetes, insulin resistance, blood pressure and MetS score) in early pregnancy.

## Methods

### Study design and participants

The present cross-sectional study utilized data from the HealthyMoms trial (clinicaltrials.gov; NCT03298555) and detailed information regarding study design and methodology has been published in the study protocol^22^. Briefly, the HealthyMoms trial is a two-arm randomized controlled trial that examines the effectiveness of a smartphone app (the HealthyMoms app) for promoting healthy weight gain (primary outcome), diet and physical activity during pregnancy. Participants were recruited at routine visits in the first trimester at maternity clinics in Linköping, Norrköping and Motala, Sweden during October 2017 to March 2020. The inclusion criteria were a singleton pregnancy, age of 18 years and above and an ability to read and speak Swedish sufficiently well to understand the content of the HealthyMoms app and provide written informed consent. In the present study, we analyzed data from the baseline measure conducted around gestational week 14 (13.9 ± 0.7 gestational weeks) before the randomization and access to any intervention content (i.e. the HealthyMoms app)^22^. A total of 305 women completed the baseline measurement that was conducted in the morning after an overnight fast. During the measurement, participants provided a fasting blood sample, had their body composition and physical fitness measured and completed questionnaires including questions regarding age, pre-pregnancy weight and physical activity level^23^, parity, occupation, and educational attainment. Of the 305 women, two were not able to perform the 6-min walk test to assess cardiorespiratory fitness (due to pelvic girdle pain and recent pneumonia) and thus the final analytic sample included 303 women. The HealthyMoms trial has received approval from the Regional Ethical Review Board in Linköping, Sweden (DNR: 2017/112-31 and 2018/262-32) and all women provided a written informed consent before entering the trial.

### Study variables

#### Body composition

Body height was measured by means of a wall-stadiometer (Tillquist, Spånga, Sweden). Body composition and weight were measured using the Bod Pod which utilize air-displacement plethysmography as previously described^24^. Briefly, air-displacement plethysmography measures body volume and together with an accurate measure of body weight, the body density can be calculated. By using appropriate densities for FM and FFM, body composition can then be calculated using the so-called two component model (i.e. dividing the body into FM and FFM)^25,26^. The use of air-displacement plethysmography can also provide accurate estimates of body composition in pregnancy if the increase in hydration of the FFM (and consequently lower FFM density) is accounted for^25,26^. Therefore, we utilized densities for FM (0.900 g/cm^3^) and FFM (1.098 g/cm^3^) appropriate for gestational week 14^26^ to calculate the % fat mass (% FM). The FFM-density appropriate for gestational week 14 was calculated using the equation by Most et al.^26^ which is based on data from van Raij^27^. BMI was calculated as weight (kg) divided by height squared (m^2^). Fat mass index (FMI) and fat-free mass index (FFMI) were calculated as FM (kg) or FFM (kg) divided by height squared (m^2^), respectively.

#### Physical fitness

Cardiorespiratory fitness was measured using the 6-min-walk test. In this test, women were instructed to walk as far as possible (back and forth in a 30-m corridor) over a period of 6 min^22,28^. The distance covered (in m) was used as a measure of cardiorespiratory fitness. Average heart rate during the 6-min walk test was assessed (Polar M400, Polar Electro Oy, Kempele, Finland) as a measure of exertion. Upper body muscular strength was measured using the handgrip test. In this test, participants squeezed an analogue dynamometer (TKK 5001, Grip-A, Takei, Tokyo, Japan) as hard as possible for a few seconds^22^. Prior to the test hand size was measured to determine grip span and the dynamometer was adjusted accordingly in order to assure correct setting to acquire maximal handgrip strength^29^. The women performed the test two times with each hand. Subsequently, the best attempt of each hand was averaged and used in the analysis.

#### Cardiovascular health

A venous fasting blood sample was taken to analyze glucose, insulin, cholesterol, triglycerides, low-density lipoprotein (LDL) and HDL cholesterol. Plasma glucose was analyzed by means of the glucose hexokinase method and serum insulin was analyzed using the Elecsys electrochemiluminescence immunoassay on a Cobas 602 (Roche Diagnostics Scandinavia AB, Bromma, Sweden). Plasma concentrations of total cholesterol, HDL cholesterol, and triglycerides were measured directly, using the enzymatic, colorimetric method on a Cobas c 701 module (Roche Diagnostics Scandinavia AB, Bromma, Sweden), while LDL cholesterol was calculated by the Friedewald equation^30^. All analyzes were performed at the Department of Clinical Chemistry, Linköping University, Linköping, Sweden, which is accredited for these analyses (ISO/IEC 17025).

Gestational diabetes was defined as fasting plasma glucose ≥ 5.1 mmol/l according to the International Association of Diabetes and Pregnancy Study Groups Recommendation^31^. Homeostatic model assessment for insulin resistance (HOMA-IR) was calculated using fasting glucose and insulin values (fasting insulin [µU/L] × fasting glucose [mmol/L])/22.5)^32^. Due to its skewness, HOMA-IR was transformed with the natural logarithm (ln) in the statistical analyses. Systolic and diastolic blood pressure were measured using an electric sphygmomanometer (ProBP 3400 series, WelchAllyn, NY, USA) after a five-min rest in an upright resting position. Two measurements of blood pressure were conducted and if either of the systolic or diastolic blood pressure differed more than 10 mmHg, a third measurement was performed. The averages of systolic and diastolic blood pressure, respectively, were used in the analysis. We also calculated a MetS score using the components in the MetS omitting waist circumference since body fatness was a main exposure in the study. Thus, the MetS score was calculated as the standardized sum of the z scores of triglycerides, inverted HDL cholesterol, glucose and the average of systolic and diastolic blood pressures as described previously^33^. A high MetS score as well as high HOMA-IR and average of systolic and diastolic blood pressure were defined as 1 standard deviation (SD) above the mean or more.

### Statistical analysis

As reported elsewhere^22^, the HealthyMoms trial was dimensioned to be sufficiently powered for the primary outcome (i.e. gestational weight gain). For the current analysis, a sample size of 303 women would provide 80% power (two-tailed, α = 0.05) to detect a standardized regression coefficient of 0.16. First, we examined associations of body composition and physical fitness with cardiovascular health variables using linear regression. Three sets of regression models were fitted: one unadjusted, one partially adjusted (including age, educational attainment [university degree vs. no other education] and parity [0 vs. ≥ 1]) and one adjusted (including covariates in the partially adjusted model as well as mutual adjustments for cardiorespiratory fitness, handgrip strength, FMI and FFMI). The adjusted model with BMI and % FM as exposures did not include FMI and FFMI (as BMI and %FM are strongly dependent of FMI and FFMI). Second, we calculated the odds ratios (ORs) of gestational diabetes and high HOMA-IR, average of systolic and diastolic blood pressure and MetS score per 1 SD difference (to facilitate comparison between exposures) in body composition and physical fitness variables by means of binary logistic regression. Three sets of regression models (i.e. unadjusted, partially adjusted, and adjusted) were generated with similar adjustments as described above. We did not observe any violations against the assumptions of our regression models^34^. Statistical analysis was conducted using SPSS (IMB SPSS statistics, version 26, IBM Corp., NY, USA) and two-sided *P* values < 0.05 (without adjustments for multiple comparisons) were considered statistically significant.

## Results

### Descriptive characteristics

Based on participants’ self-reported pre-pregnancy BMI (23.7 ± 3.9 kg/m^2^), 2.0% (*n* = 6) had underweight, 70.0% (*n* = 212) had normal weight, 21.5% (*n* = 65) had overweight and 6.6% (*n* = 20) had obesity before pregnancy. Table 1 presents the descriptive data of the women measured around gestational week 14.

**Table 1:** Descriptive characteristics of the women in early pregnancy (*n* = 303).

	Value^a^	Min–Max
General characteristics		
Age (y)	31.3 ± 4.1	20–44
Educational attainment		
Primary school (9 y)	0.7% (2)	
High school (12 y)	21.5% (65)	
University degree	77.9% (236)	
Parity		
0	57.8% (175)	
≥1	42.2% (128)	
Weight (kg)	67.6 ± 11.6	44.7–120.0
Height (m)	1.67 ± 0.06	1.46–1.82
Smoking before pregnancy	2.0% (6)	
Body composition		
BMI (kg/m^2^)	24.2 ± 3.8	17.4–41.1
FMI (kg/m^2^)	7.9 ± 3.2	3.6–22.7
FM (%)	31.8 ± 7.3	17.2–55.3
FFMI (kg/m^2^)	16.3 ± 1.3	12.8–20.0
Physical fitness		
6-min walk test (m)	671 ± 55	497–803
Handgrip strength test (kg)	33.2 ± 5.1	13.8–49.8
Cardiovascular health variables		
Glucose^b,c^ (mmol/l)	4.8 ± 0.3	3.3–5.8
Insulin^d^ (mIU/l)	6.4 ± 3.0	1.7–19.0
HOMA-IR^b^	1.4 ± 0.7	0.4–4.5
Systolic blood pressure (mmHg)	108 ± 8	91–140
Diastolic blood pressure (mmHg)	70 ± 6	54–96
Total cholesterol^c^ (mmol/l)	4.6 ± 0.7	3.1–6.9
Triglycerides^c^ (mmol/l)	1.0 ± 0.4	0.4–3.0
HDL cholesterol^c^ (mmol/l)	2.0 ± 0.3	1.1–3.0
Gestational diabetes^b^	12.3% (37)	
HOMA-IR above 1 SD of mean^e^	17.2% (52)	
Blood pressure above 1 SD of mean^e^	14.9% (45)	
MetS-score above 1 SD of mean	14.6% (44)	

*BMI* body mass index, *FM* fat mass, *FMI* fat mass index, *FFMI* fat-free mass index, *HOMA-IR* homeostatic model assessment-insulin resistance, *MetS Score* Metabolic Syndrome score, *SD* standard deviation.

^a^Values are Mean ± SD for continuous variables or % (n) for categorical variables.

^b^*n* = 302.

^c^Measured in plasma.

^d^Measured in serum.

^e^Cut-offs for 1 SD above the mean; HOMA-IR: ≥ 2.00; Average of systolic and diastolic blood pressure: 96.25 mmHg.

### Body composition and cardiovascular health

Associations of body composition with cardiovascular health variables in early pregnancy (both measured around gestational week 14) examined by linear regression are presented in Table 2. BMI, FMI and % FM were all strongly and positively associated with glucose, HOMA-IR, systolic and diastolic blood pressure and MetS score (all β ≥ 0.30, all *P* < 0.001). Noteworthy, associations with FMI were generally unaffected by the mutual adjustment for FFMI and physical fitness (i.e. the adjusted model). Higher FFMI was statistically significantly associated with higher glucose, HOMA-IR, systolic and diastolic blood pressure and MetS score in the unadjusted and partially adjusted model (all β ≥ 0.12, all *P* ≤ 0.040), although associations were weaker than the corresponding associations for BMI, FMI and % FM. Noteworthy, all associations between FFMI and the cardiovascular health variables were considerably weaker and not statistically significant in the adjusted model (all *P* ≥ 0.41), i.e. after adjustments for FMI and physical fitness.

**Table 2:** Associations of body composition with cardiovascular health in early pregnancy examined by linear regression.

Cardiovascular health variables	Body composition variables	Unadjusted		Partially Adjusted^a^		Adjusted^b^	
		β	*P*	β	*P*	β	*P*
Glucose	BMI	0.39	<0.001	0.38	<0.001	0.41	<0.001
	FMI	0.40	<0.001	0.39	<0.001	0.40	<0.001
	% FM	0.36	<0.001	0.36	<0.001	0.38	<0.001
	FFMI	0.18	0.002	0.17	0.003	0.05	0.45
HOMA-IR	BMI	0.55	<0.001	0.56	<0.001	0.57	<0.001
	FMI	0.58	<0.001	0.58	<0.001	0.61	<0.001
	% FM	0.58	<0.001	0.57	<0.001	0.59	<0.001
	FFMI	0.18	0.001	0.21	<0.001	−0.01	0.87
Systolic blood pressure	BMI	0.34	<0.001	0.34	<0.001	0.33	<0.001
	FMI	0.35	<0.001	0.35	<0.001	0.33	<0.001
	% FM	0.31	<0.001	0.31	<0.001	0.30	<0.001
	FFMI	0.15	0.009	0.16	0.006	0.03	0.68
Diastolic blood pressure	BMI	0.33	<0.001	0.34	<0.001	0.33	<0.001
	FMI	0.35	<0.001	0.36	<0.001	0.37	<0.001
	% FM	0.34	<0.001	0.34	<0.001	0.34	<0.001
	FFMI	0.12	0.040	0.13	0.029	−0.02	0.70
MetS score	BMI	0.50	<0.001	0.50	<0.001	0.51	<0.001
	FMI	0.52	<0.001	0.51	<0.001	0.51	<0.001
	% FM	0.48	<0.001	0.47	<0.001	0.47	<0.001
	FFMI	0.21	<0.001	0.21	<0.001	0.05	0.41

*β,* standardized regression coefficient, *BMI* body mass index; *FM* fat mass, *FMI* fat mass index, *FFMI* fat-free mass index, *HOMA-IR* homeostatic model assessment-insulin resistance, *MetS score* Metabolic Syndrome score.

^a^Model included age, educational attainment, and parity.

^b^Model included age, educational attainment, and parity as well as cardiorespiratory fitness and handgrip strength. For FMI and FFMI, the models were mutually adjusted for FMI and FFMI.

**Fig. 1:**
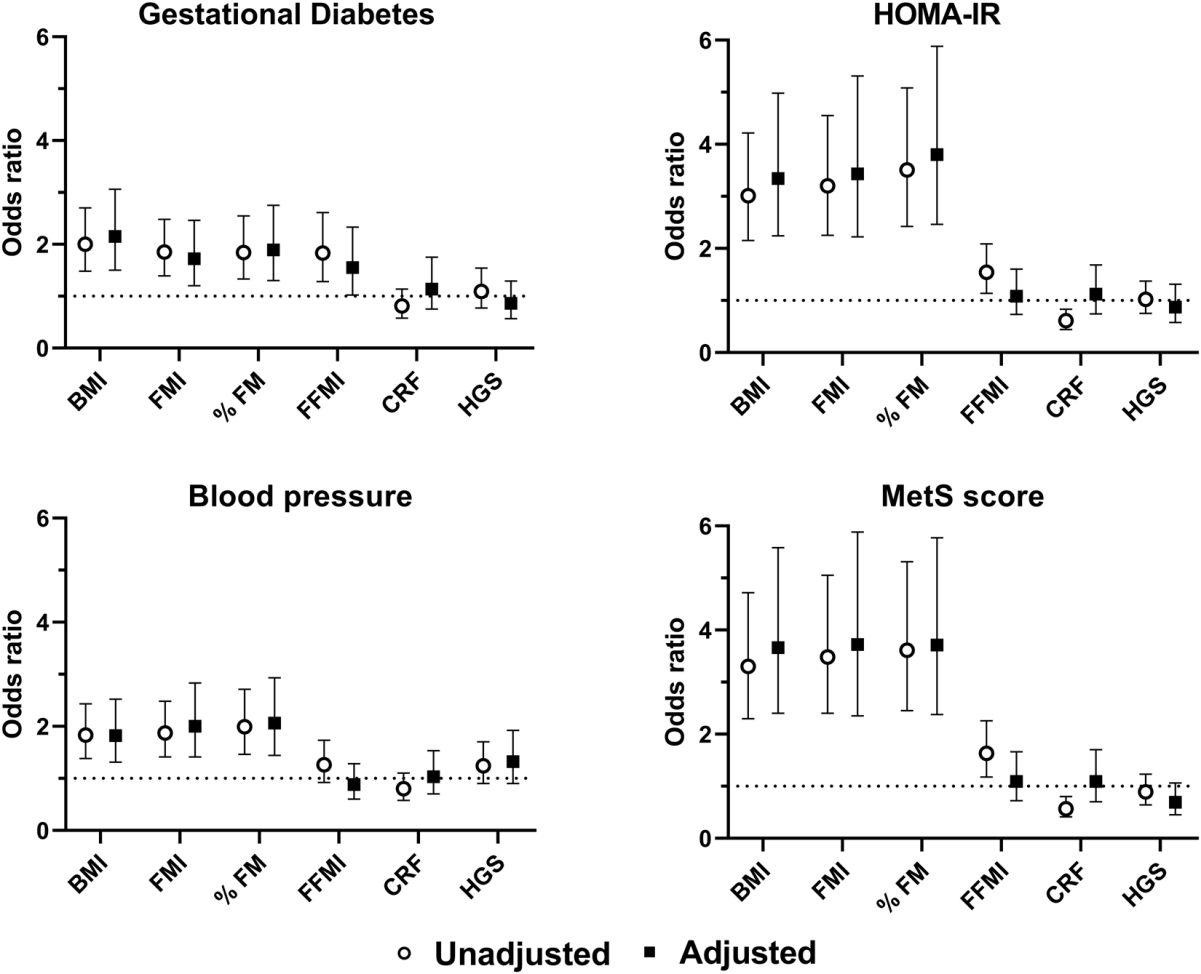
Body composition, physical fitness and cardiovascular health in early pregnancy. Odds ratios of gestational diabetes and high (defined as above 1 SD above the mean) HOMA-IR, average of systolic and diastolic blood pressure and MetS score per 1 SD difference in body composition and physical fitness variables measured in early pregnancy. Binary logistic regression was used to estimates odds ratios with 95% confidence intervals. Adjusted model included age, educational attainment and parity as well as cardiorespiratory fitness, handgrip strength, FMI, and FFMI (models with BMI and % FM did not include FMI and FFMI). *BMI* body mass index, *CRF* cardiorespiratory fitness, *FM* fat mass, *FMI* fat mass index, *FFMI* fat-free mass index, *HGS* handgrip strength, *HOMA-IR* homeostatic model assessment-insulin resistance, *MetS score* Metabolic Syndrome score.

Figure 1 shows the odds ratios of gestational diabetes and high (defined as 1 SD above the mean) HOMA-IR, average of systolic and diastolic blood pressure and MetS score associated with body composition and physical fitness variables (detailed data in Table S1). One SD higher BMI, FMI, and % FM were all associated with considerably greater ORs for gestational diabetes (ORs: 1.72–2.14, *P* ≤ 0.003) as well as high HOMA-IR (ORs: 3.01–3.80, *P* < 0.001), average of systolic and diastolic blood pressure (1.81–2.05, *P* < 0.001) and MetS score (ORs: 3.29–3.71, *P* < 0.001) in the unadjusted and the adjusted models (complete data in Table S1). One SD higher FFMI was associated with higher ORs for gestational diabetes (OR: 1.82, *P* = 0.001), high HOMA-IR (OR: 1.54–1.63, *P* < 0.006), and MetS score (OR: 1.63, *P* = 0.004) in the unadjusted and partially adjusted model. However, estimates were attenuated in the adjusted model and only remained statistically significant, yet weaker, for gestational diabetes (OR: 1.54, *P* = 0.039).

### Physical fitness and cardiovascular health

Higher cardiorespiratory fitness had a weak but statistically significant association with lower HOMA-IR, systolic blood pressure and MetS score in the unadjusted and partially adjusted model (all β ≥ −0.12, all *P* ≤ 0.033) (Table 3). However, these associations were completely attenuated and not statistically significant (all *P* ≥ 0.31) in the adjusted model that also accounted for body composition (FMI and FFMI) and handgrip strength. Handgrip strength was not associated with any of the cardiovascular health variables (all *P* ≥ 0.078).

**Table 3:** Associations of physical fitness with cardiovascular health in early pregnancy examined by linear regression.

Cardiovascular health variables	Physical fitness variables	Unadjusted		Partially Adjusted^a^		Adjusted^b^	
		β	*P*	β	*P*	β	*P*
Glucose	Cardiorespiratory fitness	−0.09	0.13	−0.09	0.13	0.08	0.20
	Handgrip strength	0.06	0.33	0.04	0.55	−0.02	0.80
HOMA-IR	Cardiorespiratory fitness	−0.20	0.001	−0.17	0.002	0.05	0.31
	Handgrip strength	0.07	0.21	0.09	0.12	0.06	0.28
Systolic blood pressure	Cardiorespiratory fitness	−0.13	0.024	−0.12	0.033	−0.01	0.92
	Handgrip strength	0.09	0.13	0.10	0.11	0.07	0.24
Diastolic blood pressure	Cardiorespiratory fitness	−0.11	0.051	−0.11	0.062	0.02	0.81
	Handgrip strength	0.09	0.13	0.11	0.078	0.09	0.13
MetS score	Cardiorespiratory fitness	−0.19	0.001	−0.18	0.002	0.03	0.65
	Handgrip strength	0.04	0.48	0.02	0.72	−0.02	0.70

*β* standardized regression coefficient, *HOMA-IR* homeostatic model assessment-insulin resistance, *MetS score* Metabolic Syndrome score.

^a^Model included age, educational attainment and parity.

^b^Model included age, educational attainment, parity, FMI and FFMI as well as mutual adjustment for cardiorespiratory fitness and handgrip strength.

One SD higher cardiorespiratory fitness was associated with lower odds of high HOMA-IR (OR: 0.60, *P* = 0.001) and MetS score (0.57, *P* = 0.001) in the unadjusted model as presented in Fig. 1 (detailed data in Table S1). Noteworthy, these associations became attenuated and not statistically significant in the adjusted model. Finally, handgrip strength was not associated with any of the cardiovascular health variables presented in Fig. 1.

### Sensitivity analyses

We conducted several sensitivity analyses to assess the trustworthiness of our findings. First, we further assessed whether adjustments for FMI was the sole reason to the strongly attenuated estimates in the adjusted model for FFMI and cardiorespiratory fitness. As shown in Table S2, estimates were strongly attenuated by adjustments for FMI which indicates that FMI mediates the associations of FFMI and cardiorespiratory fitness with cardiovascular health. Second, we examined if further adjustments for the self-reported physical activity level before pregnancy had any influence on the estimates. However, results and conclusions remained similar after this adjustment (results not shown). Third, we also performed a series of sensitivity analyses to examine to what extent low exertion and thus lower performance and heart rate during the test may influence our findings. There was data regarding average heart rate during the 6-min walk test for 290 (95.7%) of the women. As shown in Tables S3 and S4, we re-calculated our regression models only including women with an average heart rate during the test above 60% or 70% of their estimated maximum heart rate^35^. We also performed an analysis in which we included the average heart rate (expressed as percentage of estimated maximum) during the 6-min-walk test as a covariate in the models to explore whether estimates for cardiorespiratory fitness would be influenced (Tables S3 and S4). However, in both these sensitivity analyses, associations of cardiorespiratory fitness with cardiovascular health variables were quite comparable to our main results and conclusions were similar, i.e. cardiorespiratory fitness was associated with more favorable cardiovascular health although these associations were diminished in the adjusted model.

## Discussion

### Main findings

This study examined the associations of body composition and physical fitness with gestational diabetes and cardiovascular health. The main finding was that FMI or % FM measured with state-of-the-art methodology, despite strong associations, did not convey gestational diabetes risk or markers of cardiovascular health better than BMI. Furthermore, although greater FFMI had associations with greater odds of gestational diabetes as well high HOMA-IR and MetS score, associations were strongly attenuated by adjustments for FMI. Another main finding was that performance in the 6-min-walk test was associated with lower odds for gestational diabetes, and high HOMA-IR and MetS score corroborating cardiorespiratory fitness as a marker of health. However, associations with performance in the 6-min-walk test were diminished after adjustments for FMI.

### Comparison with previous studies

We observed strong associations between body fatness variables (FMI and % FM) and cardiovascular health variables which is in line with previous literature that have reported relationships of FMI and % FM with glycemia^15^ and insulin resistance^14–16^ in pregnancy. We also expand the literature by providing data regarding the associations of accurately measured body composition in relation to blood pressure and components of the MetS in pregnancy.

Regarding the FFM, the few previous studies in relation to pregnancy have shown somewhat conflicting results and have been conducted in different stages around pregnancy. For instance, Diaz et al.^17^ reported that pre-pregnancy FFMI was at least as strong predictor of HOMA-IR in pregnancy as FMI was. This is in contrast to our previous findings that showed that FMI in gestational week 32 appeared to be more strongly correlated with HOMA-IR than FFMI (*r*^2^ = 0.32 vs. 0.14)^15^. This latter finding agree relatively well with our results showing that although greater FFMI was associated with higher glycemia, HOMA-IR, blood pressure, and MetS score, associations become attenuated in the adjusted model and were markedly weaker than for the corresponding associations with FMI in all regression models. Our findings were also clear that a potential benefit from a high FFM on cardiovascular health was evidently lacking which is in line with a review of previous studies^36^. Nevertheless, it is also relevant to consider that the relatively small variation in FFMI as compared to FMI could have contributed to the generally weak association observed.

BMI was generally as strongly associated to gestational diabetes and cardiovascular health variables as FMI and % FM, which aligns well with the fact that FFMI did not have any favorable associations with cardiovascular health. These results can also be reconciled with previous studies that have found BMI to convey cardiovascular disease risk well despite not being a very accurate proxy of body fatness^18,37,38^.

To the best of our knowledge, no previous study has examined the relation of physical fitness to gestational diabetes and cardiovascular health in pregnancy. Our novel findings show that cardiorespiratory fitness is associated with lower HOMA-IR and MetS score which show the role of cardiorespiratory fitness as a marker of health also in pregnant women. However, the associations were diminished in the adjusted model showing that cardiorespiratory fitness was not an independent predictor of cardiovascular health. The lack of associations in the adjusted model could be attributed to the fact that our sample was pregnant women, that the women had relatively good cardiovascular health, the choice of fitness test or that FM may mediate at least some of the association between fitness and cardiovascular risk factors^39^. Clearly, further studies are needed to elucidate the role of cardiorespiratory fitness for the development of gestational diabetes as well as its implications on cardiovascular health in pregnant women.

### Strengths and limitations

The major strength of the study was the relatively large sample of pregnant women that was measured using accurate body composition methodology using FFM density values appropriate for gestational week 14^25,26^. Furthermore, the comprehensive measurement of the women enabled analyses that were mutually adjusted for body composition and physical fitness.

The study also has several limitations to be acknowledged. Although this is, to the best of our knowledge, the first study to examine associations of physical fitness with gestational diabetes and cardiovascular health in pregnant women, we utilized a sub-maximal measure of cardiorespiratory fitness. The distance covered and the heart rate during the 6-min-walk-test was somewhat higher than in comparable studies^40,41^ which indicate that the women in general did perform well in the test. Nevertheless, we cannot exclude the fact that participants with too low exertion in the 6-min-walk-test may have influenced our results. Although our sensitivity analyses corroborated our findings (Table S3 and S4), future studies should consider more accurate measures of cardiorespiratory fitness. Furthermore, our sample consisted to a large extent of women with high educational attainment which somewhat limits generalizability. However, we observed a wide range in the physical fitness and body composition variables and had representation across all BMI-categories although the proportion of women with overweight and obesity was somewhat lower compared to the general pregnant population in Sweden^42^. Furthermore, we adjusted our estimates for educational attainment in the partially adjusted model with minimal influence on our estimates. Finally, in lieu of well-established criteria applicable during pregnancy we classified individuals that were 1 SD above the mean to have high HOMA-IR, blood pressure, and MetS score which also represent a limitation. Finally, the fact that we cannot differentiate the body composition of the fetus from that of its mother may be considered as a limitation. Nevertheless, the contribution of FFM and FM from the fetus may be considered insignificant considering that an average fetus in gestational week 14 weighs ~100 grams (virtually only FFM)^43,44^ while the average FFM of the maternal body is ~45 kg. Furthermore, we accounted for the pregnancy-induced changes in the FFM hydration by using a FFM-density appropriate for gestational week 14^26^.

### Clinical and public health relevance

Our study provides some findings of relevance for clinical care and public health. First, BMI conveys gestational diabetes risk and cardiovascular health as good as a state-of-the-art body composition measure. This is of importance since BMI is easily measured within clinical care and our results do not motivate that additional, often costly and time-consuming, body composition measures for the identification of women with an increased risk of gestational diabetes and cardiovascular disease should be introduced. Furthermore, the contribution of FFM to gestational diabetes and cardiovascular health appears negligible when compared to the observed importance of FM. Thus, our findings support that the focus during clinical care would be on excess FM and not on levels of FFM.

## Conclusions

FMI or % FM measured with state-of-the-art methodology were strongly associated with gestational diabetes risk and markers of cardiovascular health although associations were not stronger than the corresponding ones for BMI. Finally, greater cardiorespiratory fitness was associated with lower risk of high HOMA-IR and MetS score although these associations were attenuated when accounting for FMI.

## Supplementary information

Supplementary material
