# Supplementary material for: Associations of body composition and physical fitness with gestational diabetes and cardiovascular health in pregnancy: Results from the HealthyMoms trial
Source: Nutr Diabetes. 2021 Jun 7;11:16. doi: 10.1038/s41387-021-00158-z (PMC8184768; doi:10.1038/s41387-021-00158-z)
Supplement: Supplementary file 1 — Supplementary material [file 41387_2021_158_MOESM1_ESM.pdf]

# Associations of body composition and physical fitness with gestational diabetes and cardiovascular health in pregnancy: Results from the HealthyMoms trial

Pontus Henriksson[1], Johanna Sandborg[1,2], Emmie Söderström[1], Marja H Leppänen[2,3,4], Victoria Snekkenes[1], Marie Blomberg[5], Francisco B Ortega[2,6], Marie Löf[1,2]

[1] Department of Health, Medicine and Caring Sciences, Linköping University, Linköping, Sweden

[2] Department of Biosciences and Nutrition, Karolinska Institutet, Huddinge, Stockholm, Sweden

[3] Folkhälsan Research Center, Helsinki, Finland

[4] Faculty of Medicine, University of Helsinki, Helsinki, Finland

[5] Department of Obstetrics and Gynecology and Department of Biomedical and Clinical Sciences, Linköping University, Linköping, Sweden

[6] PROFITH (PROmoting FITness and Health through physical activity) research group, Department of Physical Education and Sports, Faculty of Sport Sciences, Research Institute of Sport and Health, University of Granada, Spain.

**Corresponding author:** Pontus Henriksson, Department of Health, Medicine and Caring Sciences, Linköping University, 581 83 Linköping, Sweden. Telephone number: 013-28 10 00. E-mail: pontus.henriksson@liu.se

**Table S1**. Detailed data regarding odds ratios of gestational diabetes and high (defined as above 1 SD above the mean) HOMA-IR, average of systolic and diastolic blood pressure and MetS score per 1 SD difference in body composition and physical fitness variables.

| **Cardiovascular health variables** | **Body composition and physical fitness variables (per SD)** | **Unadjusted** | | **Partially Adjusted[1]** | | **Adjusted[2]** | |
|---|---|---|---|---|---|---|---|
| | | OR (95 % CI) | *P* | OR (95 % CI) | *P* | OR (95 % CI) | *P* |
| Gestational diabetes | BMI | 2.00 (1.48, 2.70) | < 0.001 | 2.00 (1.47, 2.71) | < 0.001 | 2.14 (1.50, 3.06) | < 0.001 |
| | FMI | 1.85 (1.39, 2.48) | < 0.001 | 1.85 (1.38, 2.49) | < 0.001 | 1.72 (1.20, 2.46) | 0.003 |
| | % FM | 1.84 (1.33, 2.55) | < 0.001 | 1.85 (1.33, 2.57) | < 0.001 | 1.89 (1.30, 2.75) | 0.001 |
| | FFMI | 1.82 (1.28, 2.61) | 0.001 | 1.82 (1.27, 2.62) | 0.001 | 1.54 (1.02, 2.33) | 0.039 |
| | CRF | 0.81 (0.57, 1.14) | 0.22 | 0.80 (0.57, 1.13) | 0.20 | 1.14 (0.75, 1.74) | 0.54 |
| | Grip strength | 1.08 (0.77, 1.53) | 0.65 | 1.05 (0.73, 1.50) | 0.81 | 0.86 (0.57, 1.29) | 0.46 |
| HOMA-IR > 1 SD | BMI | 3.01 (2.15, 4.21) | < 0.001 | 3.16 (2.22, 4.51) | < 0.001 | 3.33 (2.24, 4.97) | < 0.001 |
| | FMI | 3.20 (2.25, 4.56) | < 0.001 | 3.33 (2.31, 4.82) | < 0.001 | 3.42 (2.21, 5.30) | < 0.001 |
| | % FM | 3.51 (2.42, 5.07) | < 0.001 | 3.65 (2.48, 5.37) | < 0.001 | 3.80 (2.46, 5.87) | < 0.001 |
| | FFMI | 1.54 (1.14, 2.09) | 0.006 | 1.63 (1.19, 2.24) | 0.003 | 1.09 (0.73, 1.61) | 0.68 |
| | CRF | 0.60 (0.44, 0.82) | 0.001 | 0.63 (0.46, 0.86) | 0.004 | 1.11 (0.74, 1.68) | 0.61 |
| | Grip strength | 1.01 (0.75, 1.37) | 0.93 | 1.01 (0.73, 1.40) | 0.94 | 0.87 (0.58, 1.31) | 0.51 |
| Average of systolic and diastolic BP > 1 SD | BMI | 1.82 (1.37, 2.43) | < 0.001 | 1.85 (1.39, 2.47) | < 0.001 | 1.81 (1.31, 2.51) | < 0.001 |
| | FMI | 1.87 (1.41, 2.48) | < 0.001 | 1.90 (1.43, 2.53) | < 0.001 | 2.00 (1.41, 2.82) | < 0.001 |
| | % FM | 1.99 (1.46, 2.71) | < 0.001 | 2.02 (1.47, 2.77) | < 0.001 | 2.05 (1.44, 2.92) | < 0.001 |
| | FFMI | 1.26 (0.92, 1.73) | 0.15 | 1.27 (0.92, 1.74) | 0.14 | 0.88 (0.60, 1.28) | 0.49 |
| | CRF | 0.80 (0.58, 1.09) | 0.16 | 0.79 (0.58, 1.09) | 0.16 | 1.03 (0.70, 1.52) | 0.89 |
| | Grip strength | 1.23 (0.90, 1.69) | 0.20 | 1.30 (0.94, 1.81) | 0.12 | 1.32 (0.90, 1.92) | 0.15 |
| MetS score > 1 SD | BMI | 3.29 (2.30, 4.71) | < 0.001 | 3.30 (2.30, 4.74) | < 0.001 | 3.64 (2.39, 5.56) | < 0.001 |
| | FMI | 3.48 (2.40, 5.05) | < 0.001 | 3.51 (2.41, 5.12) | < 0.001 | 3.71 (2.35, 5.87) | < 0.001 |
| | % FM | 3.60 (2.45, 5.31) | < 0.001 | 3.65 (2.47, 5.39) | < 0.001 | 3.70 (2.37, 5.75) | < 0.001 |
| | FFMI | 1.63 (1.17, 2.26) | 0.004 | 1.63 (1.17, 2.27) | 0.004 | 1.10 (0.72, 1.66) | 0.67 |
| | CRF | 0.57 (0.41, 0.79) | 0.001 | 0.57 (0.40, 0.79) | 0.001 | 1.08 (0.69, 1.69) | 0.73 |
| | Grip strength | 0.89 (0.64, 1.22) | 0.46 | 0.86 (0.61, 1.20) | 0.37 | 0.69 (0.45, 1.06) | 0.092 |

BMI, body mass index; BP, blood pressure; CRF, cardiorespiratory fitness; FM, fat mass; FMI, fat mass index; FFMI, fat-free mass index; HOMA-IR, homeostatic model assessment-insulin resistance; MetS score, Metabolic Syndrome score; SD, standard deviation.

[1] Model included age, educational attainment and parity.

[2] Model included age, educational attainment and parity as well as mutual adjustments for cardiorespiratory fitness, handgrip strength, FMI and FFMI (models with BMI and % FM did not include FMI and FFMI).

**Table S2**. Examining the influence of adjustments of fat mass index on the associations of fat-free mass index and cardiorespiratory fitness with cardiovascular health.

| Cardiovascular health variables | Body composition variables | Unadjusted | | Partially Adjusted[1] | | Partially adjusted[1] + FMI | |
|---|---|---|---|---|---|---|---|
| | | β | *P* | β | *P* | β | *P* |
| Glucose | FFMI | 0.18 | 0.002 | 0.17 | 0.003 | 0.05 | 0.43 |
| | CRF | -0.09 | 0.13 | -0.09 | 0.13 | 0.08 | 0.19 |
| HOMA-IR | FFMI | 0.18 | 0.001 | 0.21 | < 0.001 | 0.01 | 0.80 |
| | CRF | -0.20 | 0.001 | -0.17 | 0.002 | 0.07 | 0.18 |
| Systolic blood pressure | FFMI | 0.15 | 0.009 | 0.16 | 0.006 | 0.05 | 0.40 |
| | CRF | -0.13 | 0.024 | -0.12 | 0.033 | 0.02 | 0.80 |
| Diastolic blood pressure | FFMI | 0.12 | 0.040 | 0.13 | 0.029 | 0.01 | 0.90 |
| | CRF | -0.11 | 0.051 | -0.11 | 0.062 | 0.04 | 0.52 |
| MetS score | FFMI | 0.21 | < 0.001 | 0.21 | < 0.001 | 0.04 | 0.45 |
| | CRF | -0.19 | 0.001 | -0.18 | 0.002 | 0.02 | 0.69 |

β, standardized regression coefficient, BMI, body mass index; CRF, cardiorespiratory fitness; FM, fat mass; FMI, fat mass index; FFMI, fat-free mass index; HOMA-IR, homeostatic model assessment-insulin resistance; MetS score, Metabolic Syndrome score.

[1] Model included age, educational attainment and parity.

**Table S3**. Sensitivity analyses examining different calculations of the associations of cardiorespiratory fitness with cardiovascular health variables.

| **Cardiovascular health variables** | | **Unadjusted** | | **Partially Adjusted**[1] | | **Adjusted**[2] | |
|---|---|---|---|---|---|---|---|
| | n | β | *P* | β | *P* | β | *P* |
| **Main analysis** | | | | | | | |
| Glucose | 302 | -0.09 | 0.13 | -0.09 | 0.13 | 0.08 | 0.20 |
| HOMA-IR | 302 | -0.20 | 0.001 | -0.17 | 0.002 | 0.05 | 0.31 |
| Systolic BP | 303 | -0.13 | 0.024 | -0.12 | 0.033 | -0.01 | 0.92 |
| Diastolic BP | 303 | -0.11 | 0.051 | -0.11 | 0.062 | 0.02 | 0.81 |
| MetS score | 302 | -0.19 | 0.001 | -0.18 | 0.002 | 0.03 | 0.65 |
| **Main analyses + adjustment for average heart rate during the 6-minute walk test** | | | | | | | |
| Glucose | 289 | -0.16 | 0.015 | -0.16 | 0.017 | 0.11 | 0.16 |
| HOMA-IR | 289 | -0.36 | < 0.001 | -0.34 | < 0.001 | -0.01 | 0.84 |
| Systolic BP | 290 | -0.14 | 0.038 | -0.13 | 0.054 | 0.09 | 0.24 |
| Diastolic BP | 290 | -0.19 | 0.003 | -0.19 | 0.004 | 0.00 | 0.96 |
| MetS score | 289 | -0.29 | < 0.001 | -0.29 | < 0.001 | 0.01 | 0.91 |
| **Including women with average heart rate ≥ 60 % of estimated max** | | | | | | | |
| Glucose | 247 | -0.12 | 0.063 | -0.12 | 0.057 | 0.10 | 0.14 |
| HOMA-IR | 247 | -0.25 | < 0.001 | -0.23 | < 0.001 | 0.06 | 0.30 |
| Systolic BP | 248 | -0.16 | 0.013 | -0.14 | 0.026 | 0.00 | 0.99 |
| Diastolic BP | 248 | -0.17 | 0.008 | -0.16 | 0.015 | 0.00 | 0.96 |
| MetS score | 247 | -0.23 | < 0.001 | -0.23 | < 0.001 | 0.03 | 0.63 |
| **Including women with average heart rate ≥ 70 % of estimated max** | | | | | | | |
| Glucose | 154 | -0.14 | 0.087 | -0.17 | 0.042 | 0.08 | 0.41 |
| HOMA-IR | 154 | -0.30 | < 0.001 | -0.29 | < 0.001 | 0.00 | 0.99 |
| Systolic BP | 155 | -0.17 | 0.035 | -0.16 | 0.058 | 0.04 | 0.69 |
| Diastolic BP | 155 | -0.19 | 0.017 | -0.18 | 0.026 | 0.01 | 0.95 |
| MetS score | 154 | -0.25 | 0.002 | -0.26 | 0.001 | 0.05 | 0.59 |

β, standardized regression coefficient, HOMA-IR, homeostatic model assessment-insulin resistance; BP, blood pressure; MetS score, clustered metabolic risk score.

[1] Model included age, educational attainment and parity.

[2] Model included age, educational attainment and parity as well as cardiorespiratory fitness, handgrip strength, FMI and FFMI.

**Table S4**. Sensitivity analyses examining different calculations of the odds ratios of gestational diabetes and high (defined as above 1 SD above the mean) HOMA-IR, average of systolic and diastolic blood pressure and MetS score per 1 SD difference in cardiorespiratory fitness.

| Cardiovascular health variables | n | Unadjusted | | Partially Adjusted[1] | | Adjusted[2] | |
|---|---|---|---|---|---|---|---|
| | | OR (95 % CI) | *P* | OR (95 % CI) | *P* | OR (95 % CI) | *P* |
| **Main analysis** | | | | | | | |
| Gestational diabetes | 302 | 0.81 (0.57, 1.14) | 0.22 | 0.80 (0.57, 1.13) | 0.20 | 1.14 (0.75, 1.74) | 0.54 |
| HOMA-IR above 1 SD | 302 | 0.60 (0.44, 0.82) | 0.001 | 0.63 (0.46, 0.86) | 0.004 | 1.11 (0.74, 1.68) | 0.61 |
| Average BP above 1 SD | 303 | 0.80 (0.58, 1.09) | 0.16 | 0.79 (0.58, 1.09) | 0.16 | 1.03 (0.70, 1.52) | 0.89 |
| MetS score above 1 SD | 303 | 0.57 (0.41, 0.79) | 0.001 | 0.57 (0.40, 0.79) | 0.001 | 1.08 (0.69, 1.69) | 0.73 |
| **Main analyses + adjustment for average heart rate during the 6-minute walk test** | | | | | | | |
| Gestational diabetes | 289 | 0.72 (0.49, 1.07) | 0.10 | 0.70 (0.47, 1.05) | 0.083 | 1.22 (0.71, 2.10) | 0.46 |
| HOMA-IR above 1 SD | 289 | 0.41 (0.28, 0.60) | < 0.001 | 0.41 (0.27, 0.61) | < 0.001 | 0.89 (0.52, 1.52) | 0.66 |
| Average BP above 1 SD | 290 | 0.68 (0.47, 0.98) | 0.038 | 0.68 (0.47, 0.99) | 0.044 | 1.00 (0.61, 1.64) | 0.99 |
| MetS score above 1 SD | 289 | 0.38 (0.25, 0.58) | < 0.001 | 0.36 (0.23, 0.55) | < 0.001 | 0.80 (0.46, 1.41) | 0.45 |
| **Including women with average heart rate ≥ 60 % of estimated max** | | | | | | | |
| Gestational diabetes | 247 | 0.75 (0.51, 1.11) | 0.16 | 0.74 (0.50, 1.11) | 0.15 | 1.23 (0.75, 2.04) | 0.42 |
| HOMA-IR above 1 SD | 247 | 0.51 (0.36, 0.74) | < 0.001 | 0.53 (0.37, 0.78) | 0.001 | 1.15 (0.70, 1.89) | 0.58 |
| Average BP above 1 SD | 248 | 0.70 (0.48, 1.00) | 0.050 | 0.70 (0.49, 1.02) | 0.063 | 0.90 (0.56, 1.44) | 0.65 |
| MetS score above 1 SD | 247 | 0.49 (0.33, 0.72) | < 0.001 | 0.48 (0.32, 0.71) | < 0.001 | 1.20 (0.71, 2.03) | 0.49 |
| **Including women with average heart rate ≥ 70 % of estimated max** | | | | | | | |
| Gestational diabetes | 154 | 0.70 (0.44, 1.12) | 0.14 | 0.68 (0.42, 1.09) | 0.11 | 0.98 (0.51, 1.87) | 0.95 |
| HOMA-IR above 1 SD | 154 | 0.49 (0.32, 0.76) | 0.001 | 0.49 (0.31, 0.76) | 0.002 | 0.83 (0.44, 1.54) | 0.55 |
| Average BP above 1 SD | 155 | 0.62 (0.40, 0.97) | 0.035 | 0.63 (0.40, 1.00) | 0.051 | 0.97 (0.51, 1.85) | 0.93 |
| MetS score above 1 SD | 154 | 0.45 (0.29, 0.71) | 0.001 | 0.44 (0.28, 0.70) | 0.001 | 1.14 (0.60, 2.19) | 0.69 |

β, standardized regression coefficient, HOMA-IR, homeostatic model assessment-insulin resistance; BP, blood pressure; MetS score, clustered metabolic risk score; SD, standard deviation.

[1] Model included age, educational attainment and parity.

[2] Model included age, educational attainment and parity as well as cardiorespiratory fitness, handgrip strength, FMI and FFMI.
